# Analysis of the Hosts and Transmission Paths of SARS-CoV-2 in the COVID-19 Outbreak

**DOI:** 10.3390/genes11060637

**Published:** 2020-06-09

**Authors:** Rui Dong, Shaojun Pei, Changchuan Yin, Rong Lucy He, Stephen S.-T. Yau

**Affiliations:** 1 Department of Mathematical Sciences, Tsinghua University, Beijing 100084, China; dongr15@mails.tsinghua.edu.cn (R.D.); psj17@mails.tsinghua.edu.cn (S.P.); 2 Department of Mathematics, Statistics and Computer Science, University of Illinois at Chicago, Chicago, IL 60607, USA; cyin1@uic.edu; 3 Department of Biological Sciences, Chicago State University, Chicago, IL 60628, USA; rhe@csu.edu

**Keywords:** SARS-CoV-2, COVID-19, transmission path, Natural Vector method

## Abstract

The severe respiratory disease COVID-19 was initially reported in Wuhan, China, in December 2019, and spread into many provinces from Wuhan. The corresponding pathogen was soon identified as a novel coronavirus named SARS-CoV-2 (formerly, 2019-nCoV). As of 2 May, 2020, over 3 million COVID-19 cases had been confirmed, and 235,290 deaths had been reported globally, and the numbers are still increasing. It is important to understand the phylogenetic relationship between SARS-CoV-2 and known coronaviruses, and to identify its hosts for preventing the next round of emergency outbreak. In this study, we employ an effective alignment-free approach, the Natural Vector method, to analyze the phylogeny and classify the coronaviruses based on genomic and protein data. Our results show that SARS-CoV-2 is closely related to, but distinct from the SARS-CoV branch. By analyzing the genetic distances from the SARS-CoV-2 strain to the coronaviruses residing in animal hosts, we establish that the most possible transmission path originates from bats to pangolins to humans.

## 1. Introduction

Coronaviruses (CoVs) are members of the subfamily *Coronavirinae* in the family *Coronaviridae* and the order *Nidovirales*. They can cause respiratory and intestinal infections in animals and humans [1], and are considered to be highly pathogenic to humans since the outbreak of SARS (severe acute respiratory syndrome) in Guangzhou, China in 2003 [2]. Another highly pathogenic coronavirus, Middle East respiratory syndrome coronavirus (MERS-CoV), emerged ten years after SARS and caused hundreds of fatalities. Four other types of coronaviruses which can infect humans but only cause mild symptoms are HCoV-229E, HCoV-HKU1, HCoV-NL63, and HCoV-OC43 [1]. Coronaviruses are divided into alpha-coronaviruses and beta-coronaviruses which may infect mammals, gamma-coronaviruses, and delta-coronaviruses which primarily infect birds. Among the six human coronaviruses, HCoV-229E and HCoV-NL63 are alpha-coronaviruses, while the other four HCoVs are beta-coronaviruses. The viruses were initially sorted into these genera based on serology but are now taxonomically classified by genomic phylogeny [2,3].

**Figure 1:**
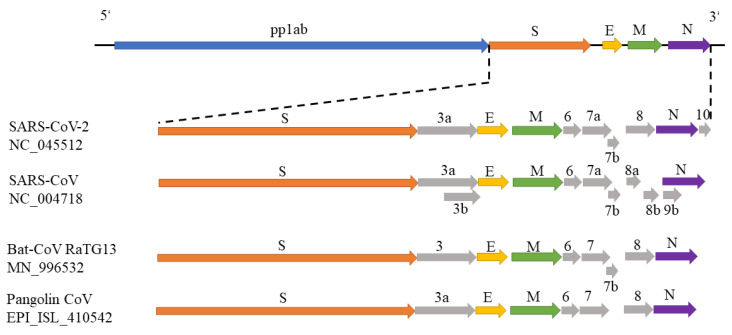
The genomic structures of SARS-CoV-2, SARS-CoV, bat-CoV and pangolin-CoV. The genomic structures of SARS-CoV-2 (NC_045512), SARS-CoV (NC_004718) and bat-CoV (MN_996532) were drawn according to their annotations in NCBI GenBank. The genomic structure of pangolin-CoV was drawn in [8].

In December 2019, a severe respiratory disease was reported in Wuhan, China, and spread in many provinces radiating outwards. Now it has become a global pandemic, which has already caused over 3 million confirmed cases and 235,290 deaths in the globe. Chinese health authorities were the first to report the complete genome of SARS-CoV-2 in GenBank (NC_045512) and annotated 11 open reading frames (ORFs) [4]. The genomic structure of coronavirus SARS-CoV-2 is shown in Figure 1, which is similar to other beta-coronaviruses. The 5’-terminal two-thirds of the genome encodes replicase polyprotein 1ab (pp1ab) with a length of 21,290 nt and contains 16 predicted non-structural proteins. The 3’ terminus encodes four structural proteins and other non-structural proteins, including spike glycoprotein (S), ORF3a, envelope small membrane protein (E), membrane protein (M), ORF6, ORF7a, ORF7b, ORF8, nucleocapsid protein (N) and ORF10 in order. Especially, the spike S protein with the Receptor-Binding domain (RBD) is the primary determinant of viral tropism and is responsible for host receptor binding and membrane fusion [5,6]. During the SARS-CoV infection, S protein first binds to the cellular receptor angiotensin-converting enzyme 2 (ACE2), and the ACE2–virus complex is then translocated to endosomes, where the S protein is cleaved by the endosomal acid proteases (cathepsin L) to activate its fusion activity [7]. Therefore, SARS-CoV-2 S protein may be the target of new SARS-CoV-2 vaccines [7].

Considering the epidemic nature SARS-CoV-2 in the COVID-19 outbreak, identifying the intermediate and original host of SARS-CoV-2 is crucial for preventing new large-scale infection and transmission of viruses. Coronaviruses can spread with direct or intermediate hosts such as avians, bats, bovines, camels, canines, civets, felines, murines, and porcines [2]. Pangolins are recently identified as possible hosts for coronaviruses [8]. It is well acknowledged that controlling first-generation infection cases has more significant effects than later person-to-person transmission stage. A series of policies such as locking down Wuhan had been established by the Chinese government with significant effects. It is therefore of great importance to identify the correct host and to cut off the transmission from animals to humans as soon as possible. Bats were suggested as the natural reservoir hosts for SARS-CoV and most coronaviruses [9,10,11], and subsequently, the discovery of SARS-related coronaviruses globally supports the connection between bats and coronaviruses. Among the intermediate hosts, masked palm civets were first considered as the host of SARS-CoV [12], while later research revealed that the coronavirus strains found in civets were transmitted from other animals [13]. On the other hand, dromedary camels were considered highly related in the case of MERS-CoV [14].

Genomic phylogenetics provides insights on the evolution and classification of viruses, and is especially important for tracking the origin of SARS-CoV-2 [15,16,17]. Based on alignment approaches, current research only associated the SARS-CoV-2 strains with SARS-CoVs. Therefore, in this study, we first apply an alignment-free approach named Natural Vector to compare 791 complete genomes of human coronaviruses and 95 SARS-CoV-2 strains collected since the outbreak in Wuhan to construct phylogenetic analysis. The analysis of protein sequences and structures of the viruses infers the relationship among coronaviruses as well. The mutations in genomes can be an indicator of the further change in protein sequences, while proteins serve as the functional units to proceed infection on animals or humans. Thus using both genome and protein information in the study could validate each other and provide us with a comprehensive understanding of the SARS-CoV-2 strains. On the one hand, the genomes include all the necessary information that a species/virus inherits and selecting a partial region would lead to a loss of information. On the other hand, proteins perform a variety of functions in almost the whole process of infection. Therefore, in our work, the phylogenetic study was based on the genomes data, and the identification of host relies on both the results from genomes and proteins.

Further, our main study is to infer the intermediate host of SARS-CoV-2 based on the coronaviruses found in various animal hosts. By calculating the distance between SARS-CoV-2 and coronavirus genomes found in animals, we may elucidate the infection chain among animal hosts and finally to humans. Notably, we also utilized the spike proteins data in the coronaviruses of various hosts.

Theoretically, it has been proved that the natural vector gives a true distance among DNA or protein sequences. Other similar researches have also utilized the spike protein sequence to identify the host of SARS-CoV-2, previous studies suggested snakes being hosts depending on condo usages [18]. However, in a later work [19], this conclusion was refuted by showing that codon usage does not determine the hosts. Our work here considers each host source as a group and applies the Hausdorff distance in mathematics to compare the coronaviruses from different hosts. The transmission path we found here was another proof of pangolins as intermediate hosts firstly proposed by [8].

## 2. Materials And Methods

### 2.1. Dataset

The phylogeny study of coronavirus was based on the comparison between SARS-CoV-2 strains and known human coronaviruses. The SARS-CoV-2 strains were downloaded from GISAID (https://platform.gisaid.org/epi3/frontend) on 23 February, 2020, which were collected from patients at the early stage of the outbreak in Wuhan, China. 731 known human coronavirus complete genomes were downloaded from NCBI directly, which consist of 29 HCoV-229E strains, 52 HCoV-NL63 strains, 34 HCoV-HKU1 strains, 153 HCoV-OC43 strains, 214 SARS-CoVs and 249 MERS-CoVs. The genomes from non-human hosts were filtered and excluded. All sequences used in this research consisted of only four nucleotides, A, C, G, T, without ambiguous ‘N’ resulted from low sequencing accuracy. We also selected 38 coronavirus genomes from recent and previous research to construct another phylogenetic tree, with the Accession Numbers listed in the tree as well.

The host identification was also based on these 95 complete SARS-CoV-2 records from GISAID. In addition, we downloaded the animal coronaviruses from NCBI GenBank. Our analysis covered the ten common animal hosts: avian, bat, bovine, camel, canine, civet, feline, porcine, and pangolin. The coronaviruses in three hosts (bat, camel, and murine) can be either alpha or beta types. Though coronaviruses found in canines were also claimed to contain both alpha and beta types, we didn’t find the reliable beta coronaviruses genomes from canines on NCBI GenBank. Therefore, we considered the CoVs in three hosts (bat, camel and murine) as different groups, giving 13 host sources as shown in the following sections. Two samples from pangolin were sequenced in Guangxi province, China in 2017, while the third was sequenced in Guangdong province in 2019. These pangolin samples were smuggled into southern China according to a source [8]. Pangolin is a popular wild animal in the Chinese market. The COVID-19 outbreak in Wuhan this time was firstly located at a so-called seafood market, but actually it was a live animal market because a variety of animals were for sale in this seafood market for a long time. We collected 823 coronaviruses with other animal hosts on NCBI and 3 pangolin-CoVs from GISAID and compared these 826 records with the 95 SARS-CoV-2 strains.

For the research based on protein data, the corresponding spike protein sequences of these 826 host-CoVs and 95 SARS-CoV-2 strains were downloaded from NCBI. Polyproteins of a coronavirus were processed by viral proteinases to yield mature proteins. Among them, 3CL proteinase performs at least eleven proteolytic cleavages within the polyprotein 1ab (pp1ab), which was considered to be the drug target [20]. The spike protein, which is critical for SARS-CoV-2 infection and differs CoV types, is responsible for ACE2 receptor binding and membrane fusion. The spike protein sequence of BetaCoV/pangolin/Guangxi/P2V/2017 was obtained in [8]. Five protein structures of human coronavirus 3CL proteinase and five structures of spike protein were downloaded from RCSB (https://www.rcsb.org/). All the accession numbers of the datasets are shown in Supplementary files S1, S2 and S3.

### 2.2. Natural Vector

Many alignment-free methods have been proposed in recent years, such as Feature Frequency Profiles (FFP) [21], Fourier-based method [22,23,24]. One important alignment-free method, named Natural Vector (NV) [25], describes a nucleotide sequence by a 12-dimensional numerical vector based on the distribution of nucleotides. The Natural Vector method has been successfully applied in the molecular evolution of bacteria and viruses [26,27,28]. Given an arbitrary DNA/RNA sequence, its natural vector can be calculated instantaneously with little computational cost, and the correspondence between the natural vector and the sequence is one-to-one. This indicates that the key information hidden in the sequence can be extracted by the Natural Vector method. Here the “key information” refers to the information that can reflect the real evolutionary pattern and therefore the corresponding distances can represent the true phylogenetic relationship. So we apply the Natural Vector approach to study the genetic relationships of coronaviruses on both genome data and protein data for a comprehensive understanding of SARS-CoV-2 strains.

Let $S=(s_{1},s_{2},\ldots ,s_{n})$ be a DNA sequence of length *n*, where $s_{i}\in \left\{A,C,G,T\right\}$. Let s[k][i] be the location of the *i*-th occurrence of nucleotide *k*. The distribution of a specific nucleotide *k* within a DNA sequence can be described by three quantities:

$n_{k}$: the number of occurrences of nucleotide *k* within the sequence.

$\mu_{k}$: the mean distance of nucleotide *k* from the first position.

$$\mu_{k}=\frac{\sum_{i=1}^{n_{k}}s\left[k\right]\left[i\right]}{n_{k}}$$

$D_{2}^{k}$: the second normalized central moment of the distribution of nucleotide *k*.

$$D_{2}^{k}=\frac{\sum_{i=1}^{n_{k}}{\left(s\left[k\right]\left[i\right]-\mu_{k}\right)}^{2}}{n\times n_{k}}$$

Therefore, the 12-dim natural vector (NV) of DNA sequence can be defined as: $\left(n_{A},n_{C},n_{G},n_{T},\mu_{A},\mu_{C},\mu_{G},\mu_{T},D_{2}^{A},D_{2}^{C},D_{2}^{G},D_{2}^{T}\right)$. By replacing four types of nucleotides to 20 amino acids: $s_{i}\in \left\{A,R,N,D,C,E,Q,G,H,I,L,K,M,F,P,S,T,W,Y,V\right\}$, Natural vector can be also applied to protein sequences. Then each DNA or protein sequence is converted to a numerical unit. Thus, using the NV representation, we can efficiently perform mathematical analysis on DNA sequences and further infer the relationship of the sequences through the corresponding natural vectors.

### 2.3. Euclidean Distance and Hausdorff Distance

The biological distance between two sequences can be expressed as the Euclidean distance between the two corresponding natural vectors in 12-dim space, as applied on many datasets [25]. Therefore, for a dataset of *n* sequences, the relationship is here described as a n×n pairwise distance matrix. The pairwise distance matrix is a symmetric distance with all positive non-diagonal elements, where element (i,j) represents the distance from *i*th sequence to the *j*th. Diagonal elements are all zero because diagonal (i,i) represents the distance from *i* to itself. By defining the distance between two vectors, we gave a reliable measurement of the similarity/dissimilarity between two DNA sequences based on the correspondence between a DNA sequence and its NV.

In the host identification, the distance between the coronaviruses from two hosts was measured by mathematical techniques as well. Here we first applied Hausdorff distance to calculate the distance between two groups of coronaviruses with different hosts [29]. In mathematics, the Hausdorff distance, named after Felix Hausdorff [30], measures how far two subsets of a metric space are from each other. It turns the set of all non-empty compact subsets of a metric space to form a metric space in its right. Let *X* and *Y* be two non-empty subsets and the Hausdorff distance d(X,Y) is defined as follows:

$$d\left(X,Y\right)=\max\{\underset{x\in X}{\sup}\underset{y\in Y}{\inf}d\left(x,y\right),\underset{y\in Y}{\sup}\underset{x\in X}{\inf}d\left(x,y\right)\}$$

The MATLAB code of calculating Hausdorff distance can be downloaded from Mathworks (https://www.mathworks.com/matlabcentral/fileexchange/26738-hausdorff-distance). Hausdorff distance satisfies the three requirements of a real distance from the mathematical perspective:

- The distance between two sets is always non-negative. The distance is zero if and only if the two sets are exactly the same.
- The direction doesn’t change the distance value, i.e., d(X,Y)=d(Y,X).
- The distance satisfies the triangular inequality, i.e., d(X,Y)≤d(X,Z)+d(Z,Y), for any *X*, *Y*, and *Z*.

Another way to measure the distance between two sets is proposed here named Center distance based on convex hulls. After the transformation from sequences to vectors in Euclidean space. Let $A=\{NV_{1},NV_{2},\ldots ,NV_{n}\}$ represent a point set of NVs of *n* protein sequences. Then the convex hull of A is defined as

$$C\left(A\right)=\left\{p|p=\sum_{i=1}^{n}\alpha_{i}NV_{i},\sum_{i=1}^{n}\alpha_{i}=1,\alpha_{i}\geq 0,1\leq i\leq n\right\}$$

Based on the definition above, we can know that a convex hull is the smallest convex set containing the given point set. For two sets of vectors, each set can be depicted by its convex hull, and the barycenter of each hull is considered as the representative of the hull. Therefore, the Euclidean distance between the two barycenters represents the average distance between two sets as well.

It is crucial to define a correct distance when studying the similarity between two groups of sequences. We showed above that Hausdorff distance is a mathematical distance, which can reflect the biological distance among the sequences with different hosts.

### 2.4. Phylogenetic Study

After obtaining the pairwise distance matrix between sequences, phylogenetic analysis was constructed firstly by constructing the evolutionary tree. FastME is a distance-based phylogeny reconstruction program that works on distance matrices [31]. FastME provides distance algorithms to infer phylogeny based on balanced minimum evolution, which is the very principle of Neighbor-Joining (NJ) algorithm. It improves over NJ by performing topological moves using fast, sophisticated algorithms [32,33,34]. We applied BioNJ, an improved version of NJ based on a simple model of sequence data [35], to construct the tree and then adjusted the tree using FigTree software (https://beast.community/figtree). The phylogenetic study visualized the distance matrix results by clustering the similar units together in the evolutionary tree.

### 2.5. Natural Graph

We drew the natural graph of SARS-CoV-2 strains and coronaviruses groups with different animal hosts as well. Natural graph was first proposed in 2015 [27]. For a dataset of *n* units, we first find the neighbor of each unit by searching for the smallest distance from each unit to any other units. During this step, we usually draw a blue arrow connecting each unit with its neighbor. This gives the result of the first-level natural graph, which also shows the closest relationships among all units. Several groups will be formed after this step, and then we find the neighbor of each group by the smallest distance between groups. The distance between group A and group B is defined as the smallest distance among all distances from any unit in group A to any unit in group B. Therefore, by finding the closest neighbor of all units in several layers (in most cases, two layers are enough), natural graph successfully classifies units into several groups in the first layer and also reveals the relationships between groups in further steps. Combining the results from phylogenetic study, one can easily understand the relationship among units and also among groups of units.

All calculation in this project was performed using MATLAB R2018b on a computer with Intel(R) Core (RM) u7-5500U CPU @2.40GHz.

## 3. Results

### 3.1. Phylogenetic Study of SARS-CoV-2

**Figure 2:**
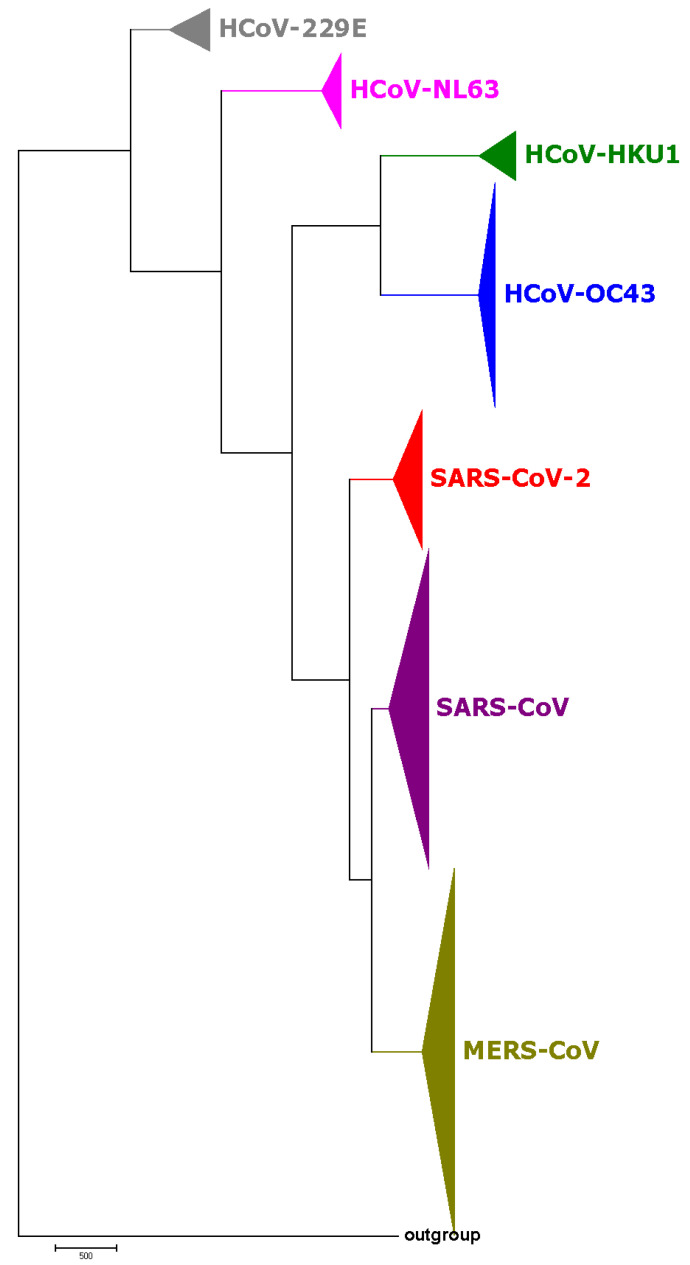
The phylogenetic tree of 95 SARS-CoV-2 and 731 known coronaviruses based on BioNJ and Natural Vector algorithm. Different colors represent different types of coronaviruses that can infect humans.

**Figure 3:**
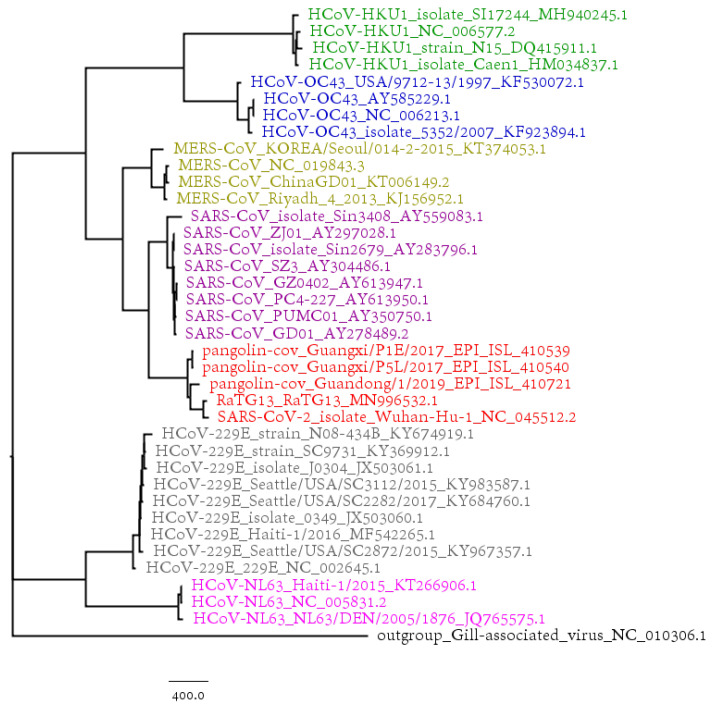
The phylogenetic tree of 38 coronavirus genomes based on BioNJ and Natural Vector algorithm.

The phylogenetic tree based on the SARS-CoV-2 strains and 731 complete genomes of human coronaviruses (HCovs) is shown in Figure 2, in which different colors represent different virus types. We also selected 38 coronavirus genomes of interest to construct the phylogenetic tree in Figure 3. Both the results in Figure 2 and Figure 3 show that the SARS-CoV and MERS-CoV are under the same branch, as the sister clade of this new SARS-CoV-2. The phylogeny indicates the close relationship between SARS-CoV and SARS-CoV-2. The branch of HCoV-HKU1, HCoV-OC43, SARS-CoV-2, SARS-CoV and MERS-CoV is for beta-coronavirus, while HCoV-229E and HCoV-NL63 are both alpha-coronaviruses.

Protein analysis was also applied to these human coronaviruses. We downloaded five crystal structures of 3CL proteinase and five crystal structures of spike protein from PDB bank. Then pairwise root-mean-square deviation (RMSD) of the structures and pairwise NV-distances of the corresponding protein sequences were calculated. The results are shown in Table 1. All the RMSD and NV-distance of 3CL proteinase and spike protein between SARS-CoV-2 and SARS-CoV are the smallest, which concurs with the classification by the complete genomes. Although current SARS-CoV-2 is in a distinct clade to SARS-CoV, previous drug study on SARS-CoV may have helpful implications for antiviral research.

**Figure 4:**
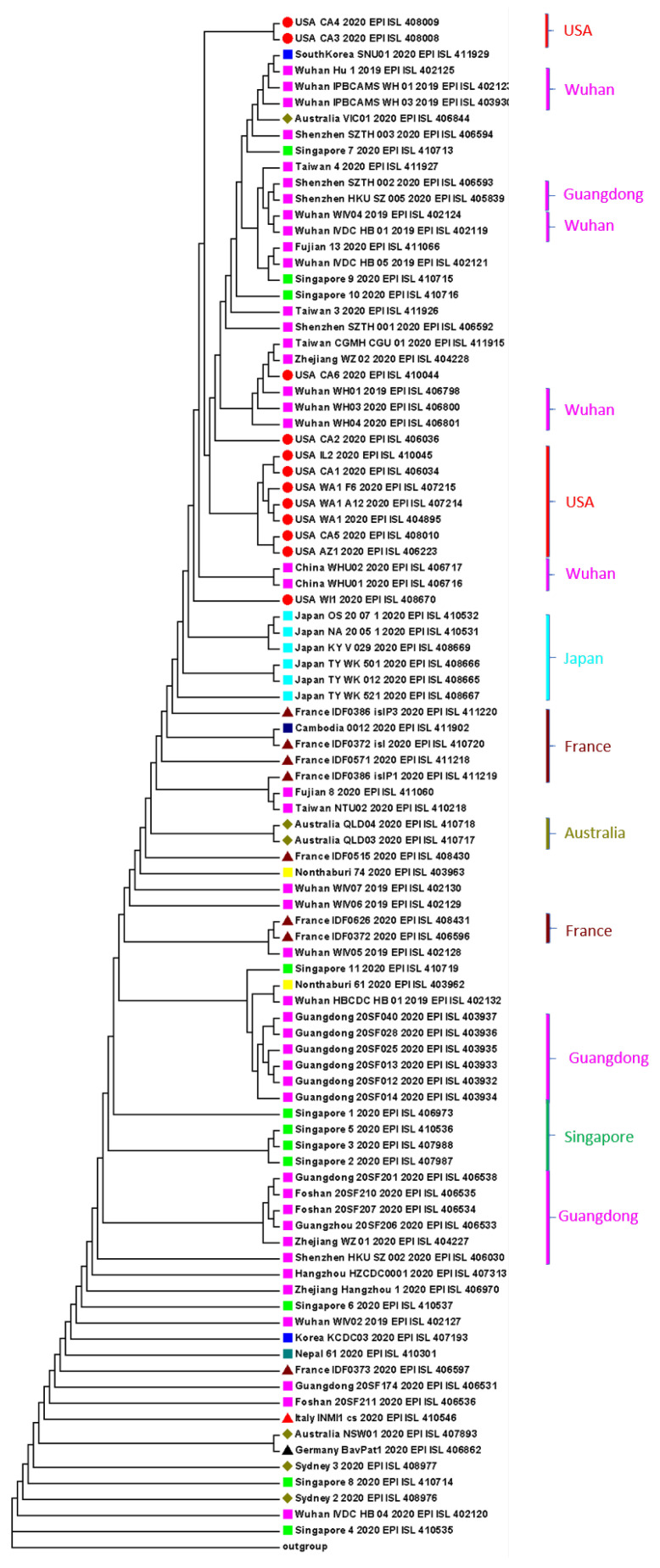
The phylogenetic tree of 95 SARS-CoV-2 strains where different colors represent the SARS-CoV-2 strains sampled from different countries.

The detailed phylogenetic tree of 95 records of SARS-CoV-2 strains is shown in Figure 4. Most of the confirmed cases had been identified in Hubei province, China, and still most of the confirmed cases had been to Hubei or related to someone in Hubei. Guangdong province, China, on the other hand, was the location of the outbreak of SARS in 2003, and the fact that both provinces have a booming market for wild animals might contribute to the outbreak. During this outbreak starting from December 2019, Guangdong also has a high number of confirmed cases at the early stage of the pandemic, though it is not geographically close to Hubei. Hence, we labeled the units in several colors in Figure 4 based on the country of each patient. From the results in Figure 4, some SARS-CoV-2 strains from the same city or province are clustered together, which indicates that the spread of SARS-CoV-2 was rapid compared to its variation speed. Therefore, the current branches are mainly formed based on the movement of populations, rather than the genome mutations. This should be emphasized in the further determination of the geographical origin of SARS-CoV-2. These 95 records were all collected at a relatively early stage, and data from afterward patients would reflect the spread of SARS-CoV-2 rather than the origin of SARS-CoV-2 strains.

Variants within the same country tend to be clustered together in Figure 4, such as all samples from Japan. This can be explained by the limited number of movement of populations between the corresponding country and China, especially Wuhan, because of the long distance and also further policies of travel restrictions. However, within China, mainly because of the tradition of returning home at the Chinese Spring Festival in the first few days when the outbreak happened, transportation was even more often than common days. Patients from the same province might be genetically distant from each other if they have different sources of infection. Patients possibly became infected during their stay in Wuhan, but showed no obvious symptoms during the incubation, and after returning to home city/province, had fever or dry cough that further confirmed to be COVID-19. In this assumption, the distance will mostly rely on their infection source in Wuhan, rather than the distance between their current locations where they were confirmed.

### 3.2. Host Identification of SARS-CoV-2

Our main work is to identify the animal hosts of SARS-CoV-2. The host identification is of essential importance to the control of virus spread and to prevent another round of emergence outbreak. The correct identification of host, source or intermediate, could lead to the effective policy to isolate humans and hosts, therefore eliminating the risk of human exposure to new viruses and limiting further transmission. During the SARS epidemic in 2003, many patients were found to have had animal exposure, especially during the early stage of the outbreak. SARS-CoV and anti-SARS-CoV antibodies were found in masked palm civets and the animal handlers in a market place, but civets are not commonly considered as the origin of SARS-CoV. Several independent groups have shown that bats are the most likely natural hosts for SARS-CoV, or found more SARSr-CoVs in bats from China, European, African and Southeast Asian countries [13,36,37,38,39,40,41,42,43,44,45,46,47,48,48]. Other animal origins of human coronaviruses include avian, bovine, camel, canine, feline, murine, porcine and pangolin [2].

**Figure 5:**
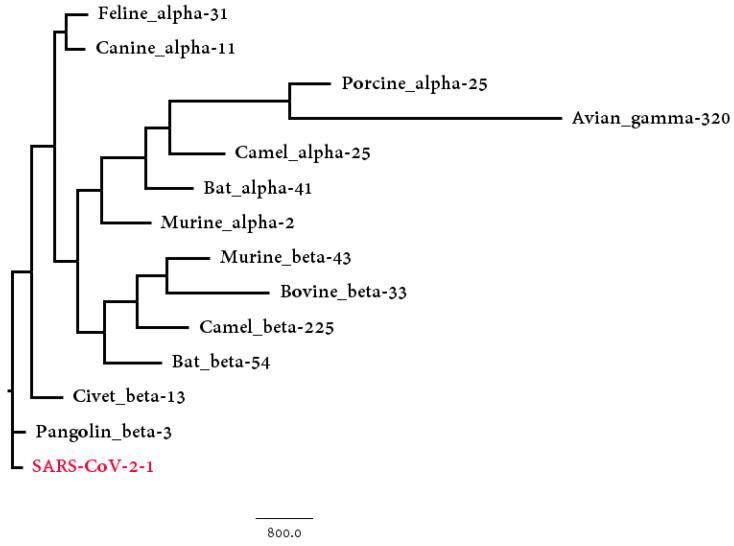
The phylogenetic BioNJ tree based on the Hausdorff distance between SARS-CoV-2 strains group and 13 possible host groups.

We collected the coronaviruses found in these animal hosts and compared them with SARS-CoV-2 strains to detect the similarity between the genome sequences. Bats, camels and murines contain both alpha and beta coronaviruses, thus they were considered as two different host groups. All together, there were 13 groups of coronaviruses found in animal hosts, and each group was then compared to the SARS-CoV-2 group, which included 95 SARS-CoV-2 strains. The corresponding phylogenetic tree based on Hausdorff distance is shown in Figure 5. Both the Hausdorff distance and Center distance between SARS-CoV-2 strains and host-CoV groups are shown in Table 2. Both results show that the beta coronaviruses found in pangolins and civets are closely related to the SARS-CoV-2 group based on the genome divergences. Though both civets and camels were considered to be intermediate hosts for other coronaviruses, the coronaviruses found in them are more distinct than the pangolin-CoVs group in Table 2.

Based on the genome data, only the coronaviruses found in four animal hosts (pangolin, civet, canine, and feline) show closer relationship to SARS-CoV-2 than bat does. The coronaviruses found in pangolins and civets are beta-coronaviruses, while the other two are alpha-coronaviruses. Thus, these four animals are possible intermediate hosts of SARS-CoV-2 but the analysis of the S protein can lead to more accurate results.

Then we analyzed the spike proteins of SARS-CoV-2 and known coronaviruses found in these animal hosts. Receptor-Binding domain (RBD) is located on the S protein and thus S protein is essential for infectivity. The protein id of spike protein of SARS-CoV-2 is YP_009724390.1. The Center distances between the spike protein of SARS-CoV-2 and the spike protein group of the coronaviruses of each host are shown in Table 2, which clearly indicates the significant similarity between SARS-CoV-2 and pangolin-CoV as well. This result coincides with the genome analysis.

**Figure 6:**
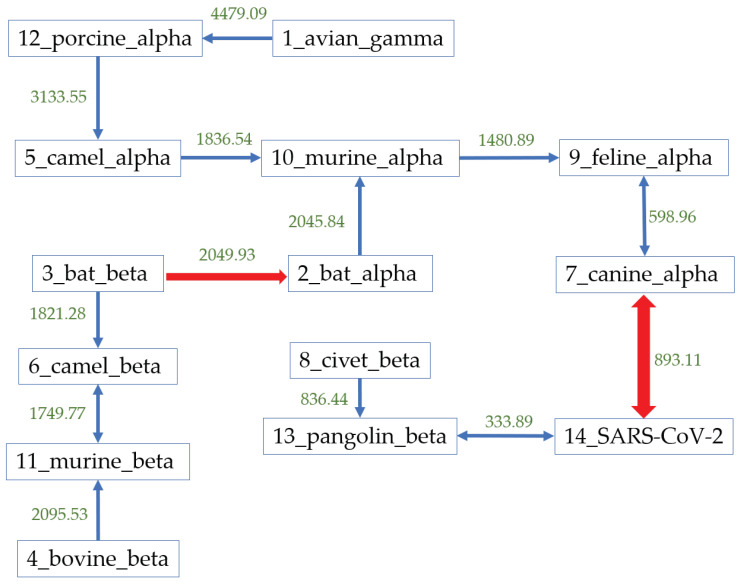
The natural graph of 13 possible host sources and SARS-CoV-2 group. The blue arrows represent the first-level relationship while the red ones represent the second-level relationships. First level indicates closer relationship.

The results agreed with each other and we further constructed the Natural Graph in Figure 6 using the Hausdorff distance based on the coronaviruses genomes. The blue arrows represent the first-level arrow. After the first-level grouping, all alpha-coronaviruses are clustered together, and the beta coronaviruses form two clusters. The green values are the corresponding Hausdorff distance between the two groups. The arrow from virus A to B means that among all viruses here, B has the smallest distance from A. The red thick arrows represent the second-level arrow, presenting the relationship among clusters that were forms from the first-level natural graph. In this case, all units are connected together after two levels. The natural graph shows that the most likely host is pangolin with the corresponding distance as 333.89. Assuming that bats are the natural reservoir of coronaviruses, pangolin should be the intermediate host of SARS-CoV-2.

Besides, in Figure 6, the coronaviruses found on many other hosts, such as civets, canines, and felines, have a relatively smaller distance to SARS-CoV-2 compared with the distance from bat-CoVs to SARS-CoV-2. Further protein analysis excludes the similarity between SARS-CoV-2 and coronaviruses found in civets, canines, and felines, according to the last column in Table 2. Therefore, combined with our results based on genomes and proteins, the natural graph indicates that the most possible path of transmission should be from bats to pangolins to humans. This transmission path is predicted from mathematical analysis, rather than biological experiments, and the determination of the natural reservoir and any intermediate host of SARS-CoV-2 requires further study and analysis.

## 4. Discussion

SARS-CoV-2 has been spreading rapidly by human-to-human transmission, and phylogenetic analysis of SARS-CoV-2 strengthens our understanding of its origin and transmission paths. Genetic relationship between SARS-CoV-2 and known coronaviruses provides insights into the host identification, and protein analysis reveals the similarity more directly because proteins are the basic functional elements in the transmission and infection process. From the phylogenetic analysis performed in this study, we confirm that SARS-CoV-2 is most similar to SARS-CoV from a range of coronavirus sequences examined, but also forms a distinct separate cluster. Thus, SARS-CoV-2 should be classified as a new member of coronaviruses, the seventh CoV member that infects humans.

The host identification found the most possible transmission chain is: from bats to pangolins to humans. Regarding this chain, we should also pay attention to civets because the distance between civet-CoV group and SARS-CoV-2 is also relatively small from genome comparison, but a little larger than bat-CoVs though protein analysis. The sequencing results from the current technology sometimes include many ambiguous letters, such as ‘N’, which would lead to inaccurate results in the transformation from DNA sequences to a numerical vector. Though we have filtered out these sequences, it also decreases the size of dataset. It is possible that laboratories can get more sequences without these ambiguous letters and therefore the distances need to be modified, which might bring back civets into our consideration. Besides, although canine-CoV is not in the same group with SARS-CoV-2 on the first level (blue arrows shown in Figure 6), the distance is within a comparable distance as 893.11. Feline-CoVs are listed 4th closest neighbor to SARS-CoV-2 group, closer to canine-CoV. Both canine-CoV and feline-CoV are alpha coronaviruses, and the recombination between alpha and beta coronaviruses are also possible in the evolution of SARS-CoV-2. If so, the close relationship between feline-CoV and SARS-CoV-2 strains might be due to the second possible chain from felines to canines to humans. This might be also another missing transmission path, but more evidence from biology is required to reach this conclusion.

In general, the limited number of the coronaviruses found in hosts, especially for pangolins, might be an issue. The calculation of natural vector is of high computational efficiency, thus once more sequencing experiments are performed and uploaded online by biological labs, we can add them into the current dataset and the results would be even more persuasive.

Meanwhile, currently available protein data of SARS-CoV-2 are mainly the primary sequences which consist of 20 amino acids, and if the structure of 11 proteins can be experimentally determined, it is possible to use the Yau-Hausdorff distance to study the relationship between protein structures [49]. Moreover, protein structures could be a powerful tool to detect protein-to-protein interaction and enhance our knowledge about the mechanics of SARS-CoV-2, therefore making it possible to develop medicines and vaccines for treating SARS-CoV-2 pathogens.

Since the outbreak of SARS-CoV-2 globally, more and more researchers have published their phylogenetic analysis using various techniques. In [50], the author collected 86 complete or near-complete genomes of SARS-CoV-2 strains on GISAID, and performed pair-wise nucleotide sequence alignment by ClustalX2. The analysis revealed 93 mutations over the entire genomes of SARS-CoV-2, located on either coding or non-coding regions, in contrast to our analysis of extracting the key information in the whole genome. On the other hand, Liu et al. and Anderson et al. have done research on the alignment of protein sequences from different sources [19,51], and they concluded that other than pangolins, snakes and turtles may also act as the potential intermediate hosts transmitting SARS-CoV-2 to humans. The host identification would always be updated based on new findings on experimental data, and we are also working on similar projects. The most significant difference between this work and other research is that we consider the coronaviruses found in each animal host as a single group and study the relationship between groups using mathematical techniques.

The result in [52] coincided with our results that pangolins are the probable zoonotic origin of SARS-CoV-2 outbreak. Despite the similar conclusion, their statement of the similarity between Pangolin-CoV and SARS-CoV-2 is 91.02% is based on alignment. In [53], the alignments of the spike surface glycoprotein receptor binding domain revealed four times more variations in the bat coronavirus RaTG13 than in pangolin-cov compared with SARS-CoV-2, suggesting the pangolin as a missing link in the transmission of SARS-CoV-2 from bats to human. Our approach provides a well-defined measurement of the distance between two groups of sequences, and both genomic and protein data suggested that pangolins are the most likely intermediate host of SARS-CoV-2. Some other research [5,6] focused on the phylogenetic analysis of spike protein data as well, which also provided insight into the study of the interaction with antiviral drugs.

## Figures and Tables

**Table 1:** The RMSD and NV distance between 3CL proteinase (6LU7)/spike protein (6VXX) of SARS-CoV-2 and the counterpart proteins of other human coronaviruses.

**3CL Proteinase**	**SARS-CoV-2**	**SARS-CoV**	**HCoV-229E**	**MERS-CoV**	**HCoV-NL63**
PDB-Number	6LU7	3AW0	2ZU2	5WKJ	6FV2
RMSD		0.72	1.10	1.53	1.25
NV-Distance		22.61	117.82	140.59	118.98
**Spike Protein**	**SARS-CoV-2**	**SARS-CoV**	**HCoV-229E**	**MERS-CoV**	**HCoV-NL63**
PDB-Number	6VXX	5X58	6U7H	5X5F	5SZS
RMSD		1.74	2.21	3.20	2.71
NV-Distance		235.39	349.86	289.54	401.99

**Table 2:** Distance from SARS-CoV-2 group to the coronavirus group of each host.

Host	Number	Hausdorff Distance ${}^{1}$	Center Distance ${}^{2}$	S-Protein Center Distance ${}^{3}$
Pangolin_beta	3	333.89	230.11	117.39
Civet_beta	13	928.40	952.39	220.21
Bat_beta	54	2400.72	1102.03	205.47
Murine_beta	43	2620.43	2358.63	254.27
Camel_beta	225	2464.57	1307.54	353.01
Bovine_beta	33	2571.48	2377.34	317.78
Avian_gamma	320	8788.62	2753.43	426.91
Bat_alpha	41	3340.69	2494.54	257.71
Camel_alpha	25	3065.11	3044.61	405.31
Canine_alpha	11	893.11	947.82	485.56
Feline_alpha	31	1205.55	107.93	453.74
Murine_alpha	2	2168.66	2125.31	482.57
Porcine_alpha	25	4981.11	2784.12	391.29

${}^{1}$ Hausdorff distance from SARS-CoV-2 strains to the coronaviruses found in each host group. ${}^{2}$ Center distance from SARS-CoV-2 strains to the coronaviruses found in each host group. ${}^{3}$ Center distance from the S protein sequences of SARS-CoV-2 strains to the S protein sequences of coronaviruses found in each host group.

## Supplementary Materials

The following are available online at https://www.mdpi.com/2073-4425/11/6/637/s1, Supplementary file S1, Supplementary file S2, and Supplementary file S3 (the newick file of Figure 2).
